# Genotyping and Bio-Sensing Chemosensory Proteins in Insects

**DOI:** 10.3390/s17081801

**Published:** 2017-08-04

**Authors:** Guoxia Liu, Philippe Arnaud, Bernard Offmann, Jean-François Picimbon

**Affiliations:** 1 Biotechnology Research Center, Shandong Academy of Agricultural Sciences, Jinan 250100, China; girlgx@sina.com; 2 Protein Engineering and Functionality Unit, University of Nantes, 44322 Nantes, France; philippe.arnaud@univ-nantes.fr (P.A.); bernard.offmann@univ-nantes.fr (B.O.); 3 QILU University of Technology, School of Bioengineering, Jinan 250353, China

**Keywords:** biotype imprinting, lipometer, mutation sensor, RNA editing, genosensing

## Abstract

Genotyping is the process of determining differences in the genetic make-up of an individual and comparing it to that of another individual. Focus on the family of chemosensory proteins (CSPs) in insects reveals differences at the genomic level across various strains and biotypes, but none at the level of individuals, which could be extremely useful in the biotyping of insect pest species necessary for the agricultural, medical and veterinary industries. Proposed methods of genotyping CSPs include not only restriction enzymatic cleavage and amplification of cleaved polymorphic sequences, but also detection of retroposons in some specific regions of the insect chromosome. Design of biosensors using CSPs addresses tissue-specific RNA mutations in a particular subtype of the protein, which could be used as a marker of specific physiological conditions. Additionally, we refer to the binding properties of CSP proteins tuned to lipids and xenobiotic insecticides for the development of a new generation of biosensor chips, monitoring lipid blood concentration and chemical environmental pollution.

## 1. Introduction

Insects represent an important agricultural problem and are one of the emerging or re-emerging causes of major epidemic diseases affecting many countries around the world. Current strategies for insect pest control are largely based on parasitoid predators, pathogens, pheromones, pheromone lures, insecticide-trapped log lures and extensive spraying of toxic insecticide chemicals or a combination of all, most notably through so-called Integrated Pest Management (IPM) programs. IPM for insect control attracts particular attention nowadays with increasing concerns about human health and environment protection, but must face several challenges, particularly the need for innovations that can counteract the adaptation mechanisms underlying insecticide resistance [1].

Identifying the insect pest is the first step before to setting up any control strategy. This is mandatory, for instance, in the control of the sweetpotato whitefly, *Bemisia tabaci*, which has a large variety of multiple strains and biotypes [2,3]. Among these biotypes, the B and Q ones happen to be the most invasive and destructive forms of *Bemisia*, with known notable differences in host selection, reproductive performance and insecticide resistance [2,3,4,5,6]. The Q biotype is particularly well known for its extremely high resistance capacity against xenobiotic neonicotinoids and a panoply of new insecticide chemicals such as nitenpyram, pymetrozine, sulfoxaflor, and cyantraniliprole. This would explain its cosmopolitan distribution and facile establishment on field crops [7]. Identification of the biotype is therefore a crucial fundamental and unavoidable step to design the selection of insecticides for control of a particular insect pest species such as *B. tabaci*.

This problem of strain-specificity in insecticide resistance is not only found in green agricultural environmental pest species, but concerns also food storage insect pests such as the red flour beetle, *Tribolium castaneum* Herbst (Coleoptera, Tenebrionidae) [8,9,10,11]. This is particularly important since fitness costs and fecundity can change with exposure and development of resistance to specific insecticide chemicals [12]. Also, for obvious other prospects, identification and selection of specific strains is similarly important in “beneficial” insects such as the domesticated silkworm moth, *Bombyx mori.* In the corridors of the Silk Road, there are thousands of genotypes, hundreds of geographical bivoltine monovoltine races and dozens of genetically modified strains that could be optimized for high production of the cultural treasure silk [13].

Here, we write a 3-part overview of insects about the genes, RNA and proteins of a very peculiar single gene family, the chemosensory protein (CSP) gene family, with a main objective to describe some of the numerous applications that can follow its study, in particular in an in-depth function and genetics analysis. We aim at providing a synthetic review of the use of chemosensory proteins as target for genotyping/bio-sensing insects with the aim of reaching the level of the biotype, such as the ones in *B. tabaci*. Most of the works come from Liu et al. who characterized the biotype of *Bemisia* based on *CSPs* but failed to put it in a more global scientific context [14,15,16], so we have collected the set of genomics data obtained in *Bemisia* and expanded it to *Tribolium* after *Bombyx*, revealing some evolutionary traits of the CSP gene family in insects. This approach is interesting because it turns out that *CSPs* are rich in genetic and epigenetic biomarkers that could be particularly useful, not only in strain identification, but also in testing a specific physiological system for mutations [14,15,16]. The capacity of a single species to exhibit an increasing number of genotypes with variable phenotypes in many widely different environments is common in insects, leading to natural mutant strains particularly well adapted to environmental changes. Therefore, genotyping and bio-sensing certainly need a new gene-focused approach in particular in our modern times of global climate change, intense crop production, air/water contamination and toxic compound accumulation.

In addition, the multifunction, clearly established for this protein family in the sweetpotato whitefly *B. tabaci* [16], strongly suggests connection between CSP binding sites and multiple biosensor chips that could become essential for biomedical, environmental and toxicological research. The most interesting point in the CSP study of insects is maybe what it could bring in regards to current approaches in biochips. Importantly, CSP-based biochips may have novelties compatible not only with mutation sensing, but also with detection of biomolecules as diverse as lipids and insecticide chemicals. Our present focus on *CSPs* explores the remarkable diversity of RNAs and protein structures provided by a multifunction gene family in light of strain biomarkers and base pair mutation probes, as well as detection and monitoring of potentially toxic foreign chemicals even at ultra-trace levels, drastically expanding the possible acute vision of a basic clean, green and safe bio-environment.

## 2. Intron Variation and Retrotransposition in a Conserved Gene Family

There are several parameters to look at in order to determine what is a good sensor or a good genotyping target. This includes notably genes that are well conserved over the course of evolution, i.e., a large family with several member genes across various organisms. The conservation of the size of the gene family and the clusters that genes form may eventually be a good indicator of evolutionary rates, if we assume that all genes with family size conservation evolve more slowly than genes without family size conservation [17]. Conservation of gene family and gene clusters certainly indicates that it is highly conserved but not necessarily the rate of evolution in this family. What could help is if there are different sizes and/or differences in the sequences that can be related to function or multi-function. In this respect, the CSP gene family is an excellent model of study for genotype and association of genes with multiple functions [16,17].

Genes that are found in large numbers, enough to be on the same chromosome as a physical cluster of several genes to be considered as a track of evolved family, usually offer a track of molecular evolution and diversification of gene function, which is essential for phylogenetic biotyping on long and short evolutionary time scales [18]. This also includes genes with low inter-individual variations to maximize the capacity of detecting differences only between biotypes (inter-biotype variation > intra-biotypes inter-individual variation). They must be present and similar in populations from different geographical regions, allowing the design of universal biotype sensor and not regionally-dependent sensor. Finally, new methods of genotyping/sensing should take into account potential variation in the characteristics targeted. This is performed nowadays using polymerase chain reaction (PCR), quantitative PCR, going fluorescence-Förster Resonance Energy Transfer (FRET) and high-throughput Next Generation Sequencing [19,20,21,22,23,24], but the sophistication, diversification and development of new next generation biotyping techniques call for having a universal tool that works for a wide range of possible changes or mutations. It applies completely to the family of chemosensory protein (CSP) genes, for which gene clusters, functional properties, regulatory traits, ontogenetic and tissue-distribution profiles as well as numerous mutations of mapped sites have been clearly established ([14,15,16,25,26,27,28,29] and this review).

**Figure 1:**
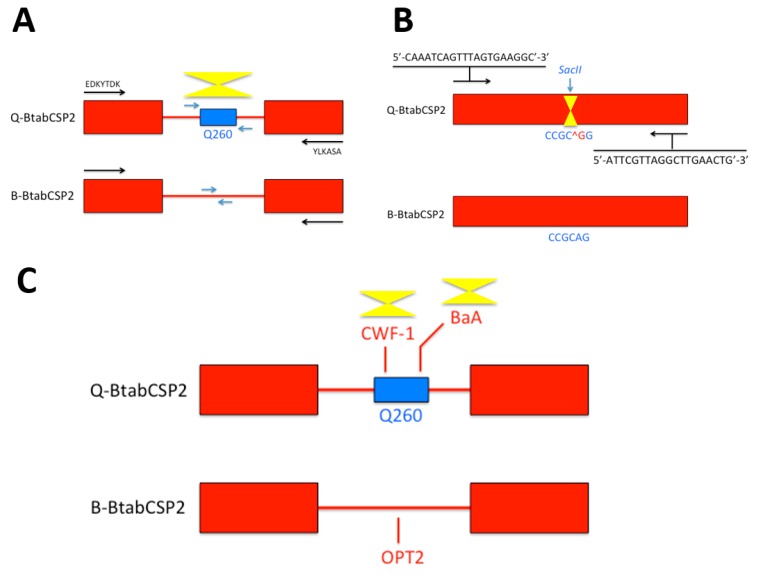
Genotyping B and Q *Bemisia* CSPs based on Q260, SacII sites and SCAR markers. (**A**) Recognition of Q-biotype by probing a Q-specific 260 bps-fragment of *BtabCSP2* intron [15]. Using genomic DNA as template, polymerase chain reactions are tuned to N- and C-termini (arrows in black) before selective amplification of Q260 fragment (in blue); (**B**) Recognition of Q-biotype by restriction enzyme digestion of PCR products encoding BtabCSP2. CCGC^GG: *SacII* restriction enzyme cleavage site (Q-biotype). The *SacII* cleavage site is absent in B-biotype (^G > A switch) [15]; (**C**) Recognition of Q-biotype by amplification of CWF-1 and BaA SCAR markers in Q260 region from *BtabCSP2* gene. B-biotype-*BtabCSP2* is more characterized by OPT12 SCAR marker (LIME) [15]. Biotyping markers for Q are underlined by yellow triangles.

Chemosensory protein (CSP) is a family of small soluble 4Cys proteins largely described in insects for expression of many various physiological systems from tissue development to immunological response [14,15,16,25,26,27,28,29]. Their occurrence not only in insects but also in bacteria emphasizes further a multifunction mainly in relation with the metabolism of xenobiotics as described in detail in the sweetpotato whitefly *B. tabaci* [16,30]. Interestingly, in a new genetic study analyzing B and Q biotypes of *B. tabaci*, a set of *chemosensory protein* (*csp*) genes were identified as molecular markers to predict the *Bemisia* biotype [14,15,16]. Identifying genomic DNA sequence encoding for *B. tabaci* chemosensory protein type 2 (BtabCSP2) revealed significant differences between the biotypes B and Q (Figure 1).

A Q260 sequence was found specifically in the intron of *BtabCSP2* in Q, not in B, urging the development of specific nucleotide primers probing against CSP intron for biotype recognition (Figure 1A) [15]. Additionally, a further comparison of CSP2 gene sequences between B and Q biotypes of *B. tabaci* revealed another biotyping method based on a specific restriction enzyme (SacII) that cuts at CCGC^GG site on BtabCSP2 in Q, but not in B. This offered a new strategy of *Bemisia* biotyping eventually based on a Cleaved Amplified Polymorpic Sequences (CAPS) method (Figure 1B) [15]. Analyzing CSP genes in B and Q, we also identified some Sequenced Characterized Amplified Region (SCAR) markers that could be used as a third strategy for insect genotyping based on *CSPs* (Figure 1C) [15]. SCAR markers specifically linked to β gene have been developed for biotyping in fruits and plants [31,32]. SCAR markers are also known to distinguish between several insect biotypes as described in the human malaria vector *Anopheles diru*, the Asian long-horned beetle *species name,* the cotton bollworm *Heliothis armigera* and the cereal aphid *Rhopalosiphum padi* L. [33,34,35,36]. In our study, we find that SCAR markers such as BaA and CWF-1 (AY841800, DQ174543) are linked to Q-BtabCSP2 gene, while B-BtabCSP2 only has OPT12 SCAR marker (EU660887). Such a specificity observed in SCAR sequence motifs linked to *CSP* is likely to help develop a new way of genotyping, particularly in the sweetpotato whitefly *B. tabaci* (Figure 1C) [15,37]. BaA, CWF-1 and OPT12 should be investigated in detail, particularly because such specific SCARs seem to associate with insecticide resistance not only in whiteflies, but also in other insect species such as the parasitoid jewel wasp *Nasonia vitripennis* [15,38]. Thus, it can be expected that the approach to *CSP* and SCAR sequence such as BaA, CWF-1 and OPT12 will help develop novel insect biotyping methods applicable in a very general sense. If they associate with insecticide resistance, then biotyping using SCARs in intron of *CSPs* may apply not only to agricultural pest species or other parasitoid wasps, but also to a wide variety of etiologic agents of human diseases, including the most highly insecticide-resistant strains in *Aedes* and *Anopheles* mosquito species.

The finding of biotype-specific distribution of SCARs in *B. tabaci* poses the question of whether short interspersed elements (SINEs) and other retrotransposons can be used in a general manner for strain or race identification in association/relation with CSP genes as markers. Retroposons are routinely used for species and/or individual strain recognition in various organisms from plants to insects [39,40,41]. It is therefore of a particular interest to note that different types of microsatellite repeat sequences, transposons and/or retrotransposons such as Bm1, Bm2, BMC1, BmRTE, L1BM, Kendo and Taguchi can be found in many CSP genes of the silkworm moth *B. mori* [29]. In particular, there is a major group of *B. mori* CSP genes (*BmorCSP1, BmorCSP4, BmorCSP6, BmorCSP9, BmorCSP10, BmorCSP13* and *BmorCSP15*) associated to SINE Bm1, while a second group of *BmorCSPs* is rather characterized by L1Bm/BMC1 transposon insertion [29]. Therefore, not only SCARs but also retroposons such as Bm1 and L1BM/BMC1 appear to be very relevant biomarkers to label specific groups of *CSPs* in a determined chromosomal region of the insect [29]. Common retroposon is indicative of common ancestry rather than common function. Similarly, *BmorCSP14*, *BmorCSP17* and *BmorCSP16* (truncated gene) share same stop codon (TAG) and probably thereby common molecular ancestry [29]. All other *BmorCSPs* retain TAA stop codons, indicating evolution of moth CSP genes by A-to-G substitution and/or shift of stop codons as described for recoding events in ciliate and prokaryotic gene families [29,42,43]. Curiously, in *Bombyx*, some multiple copies of the same type of transposon or retrotransposon have inserted within the same CSP gene (*BmorCSP4*: Bm1/Bm2; *BmorCSP7*: microsatellite repeat sequences; *BmorCSP10*: Kendo-Kendo-Bm1/Bm1; *BmorCSP17*: L1Bm repeats) [29]. These repeats or copies of the same retroposon in the same CSP gene may be indicative of internal gene duplication and intron gain during the evolution of *CSPs*, particularly in the silkworm moth *B. mori* [29,44]. Interestingly, *BmorCSP4* and *BmorCSP10* contain the same Bm1 retrotransposon insertion in intron 1 and intron 2, suggesting that the organization with three exons and two introns of *CSPs* comes from exon-intron genomic DNA structure that is duplicated upon transposition of specific insertion elements [29,45]. This view of *CSP* evolution is emphasized by the position of intron 2. Intron 2 is inserted at the codon that codes for Arg87 in *BmorCSP4*, while it is inserted at the codon that codes for Ser115 in *BmorCSP10* [29]. This is perhaps one further very sound argument to say that three exons-two introns CSP genes do not originate from the duplication of the same common ancestor, but rather from transposition and exon-intron duplication events that occurred independently in the two genes during the course of evolution. It is very likely that retroposons and retrotransposons have played a major role in generating some genetic variability in the CSP gene family, thereby offering the development of new strategies for insect biotyping at least in the silkworm moth *B. mori* [29].

We thus describe the CSP genes in the red flour beetle *Tribolium castaneum* to check for the role of retroposons in the expansion and evolution of this gene family in another insect species in order to discuss further about the use of CSP retrotransposon-based methods for future investigations in insect biotyping. This may go much beyond *Bombyx* or *Tribolium* CSPs and suggest new tools potentially universal between species.

**Figure 2:**
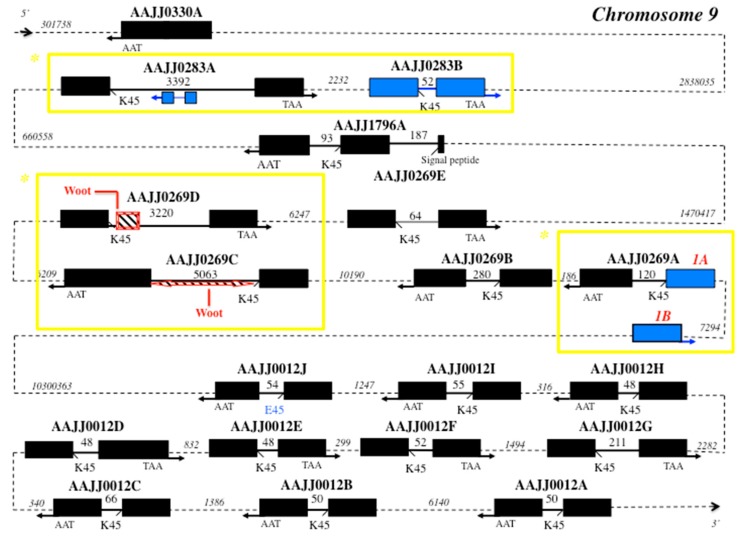
Genomic organization of CSP genes in *T. castaneum* (chromosome 9). Genes are described in Table S1. Black boxes: exons, bold plain lines: introns, dotted lines: intergenic intron regions (*length* in bps). K45 indicates intron position at the level of lysine residue at position 45. E45 (in blue) indicates lysine-to-glutamic acid switch in the exon/intron boundary at position 45 in *AAJJ0012J.* The arrow indicates the 5′–3′ (right) or 3′–5′ (left) orientation of the gene. In blue shows particular genetic markers for *Tribolium* CSPs: inverted duplicated gene in *AAJJ0283A*, duplicated exon in *AAJJ0269A*, and Woot retroposon in *AAJJ0269C* and *AAJJ0269D*. The position of Woot retroposon is indicated by a bevel box with the diagonals (downward), showing the orientation of the retroposon in *AAJJ0269C* and *AAJJ0269D*: 5′–3′. The yellow squares pinpoint potential strain-specific regions for biotyping in Coleoptera (*).

Interestingly, comparing CSP gene structures between moths and beetles, we find that the number of intronless genes is the same, but the number of three exons-two introns genes is superior in moths (Table S1, Figure 2) [29]. Analyzing the red flour beetle *T. castaneum* genome (beetlebase) [46], we find that it has a number of functional CSP genes similar to that of the lepidopteran silkworm moth *B. mori* (nineteen versus seventeen). In silico analysis of the *Tribolium* genome identified nineteen coding CSP genes from five different scaffolds (AAJJ0012, AAJJ0269, AAJJ0283, AAJJ0330 and AAJJ1796; Table S1, Figure 2). Out of the nineteen *T. castaneum* CSP genes identified, one gene is intronless (*AAJJ0330A*), one gene has three exons-two introns (*AAJJ1796A*), while the other seventeen genes all contain a single intron in general of short size (<100 bps) (Figure 2). There is an additional *TcasCSP* sequence, which is a truncated gene, similarly to *BmorCSP5*, *BmorCSP16* and *BmorCSP18* [29]; exon1 of *AAJJ0269A* exists as two nearly identical copies (Exon1A and Exon1B), the two copies being separated by only about seven thousands base pairs (Figure 2). The intronic sequences immediately flanking Exon1A and Exon1B are identical, strongly indicating that the entire Exon1 of *AAJJ0269A* was duplicated as part of recent genome duplication. It eventually makes this region of interest for strain biotyping in *Tribolium* (Figure 2). Similarly, the genes *AAJJ0269C* and *AAJJ0269D* from *Tribolium* are found 24 kb apart and the gene *AAJJ0269E* is found 24 kb apart from *AAJJ0269D* (Figure 2). This is a clear further illustration of the role of genome duplication in promoting evolutionary innovation and/or functional diversification within a specific gene family, thereby producing new traits and biomarkers in various kinds of insects, particularly in beetles. Such genome duplication has certainly contributed to the adaptive development of new beetle genotypes, strains or species [47,48], which could be investigated by sequencing various genomes in the family Tenebrionidae. Curiously, the large intron of the gene coding for *AAJJ0283A* contains at position 859-1383 a copy of *AAJJ0283B* gene arranged in an inverted orientation 3′–5′ (Figure 2). The exon sequences of these two copies of *AAJJ0283B* are identical but their intron sequences are totally different, indicating that not only genome duplication but also inverted duplication of exons have occurred in flour beetles (Figure 2). Inverted duplication and allelic variation is known for specific mouse loci [49]. All together, this would suggest an additional marker to distinguish between different various genotypes of *tribolium*. Most typical strains would carry only *AAJJ0283A*, while some other various more atypical strains would carry also the gene inversion and thereby allelic variation as described in mice [49].

Retrotransposition and inverted duplication suggest prevalence of intron gain over intron loss in the evolution of CSP genes in the two largest orders of insects, commonly the Coleoptera and the Lepidoptera [50,51,52]. However, analyzing CSP genes in *Bombyx* and *Tribolium* shows clear genetic differentiation between Coleopteran and Lepidopteran species (Figure 2) [29]. This cannot be used to edge a universal approach of biotyping, but this can be useful knowledge for a novel approach to the problem of Coleoptera- or Lepidoptera-specific genotyping. In contrast to *BmorCSPs* [29], *TcasCSP*s all have TAA stop codons, which may represent an ancestral state later converted to TAG ending codon in the order Lepidoptera. In Coleoptera, the intronless CSP gene *AAJJ0330A* sits first 2838035 bps aside the different clusters of the other CSP genes in duplication order (Figure 2). Interestingly, *AAJJ0283A* gained intron following exon duplication of *AAJJ0283B.* Intron1 in *AAJJ1796A* inserted within the nucleotide region coding for the signal peptide as found for *BmorCSP19*, possibly indicating common origin and function for these two genes (Figure 2) [29]. For these sequences, such as *AAJJ1796A* and *BmorCSP19,* which are descendent from the same common sequence in an ancestor, a molecular study can be done to develop a biomarker potentially universal between species.

Also interestingly, *TcasCSP* genes show evidence of intron gain via transposon insertion similarly to *BmorCSPs* (Figure 2) [29,53]. The two neighboring *Tribolium* CSP genes *AAJJ0269C* and *AAJJ0269D* harbor a retroposon Woot in the 5′ end or both sides of the intron (Figure 2) [53], suggesting that biotyping based on both CSPs and retroposons can be used not only in Lepidoptera, but also in some other insect orders including the Coleoptera.

In the next juxtaposed clustering, *AAJJ0012A*, *AAJJ0012B*, *AAJJ0012C*, *AAJJ0012D*, *AAJJ0012E*, *AAJJ0012F*, *AAJJ0012G*, *AAJJ0012H*, *AAJJ0012I* and *AAJJ0012J* have clearly arisen by much more recent gene duplication, which makes them not so suitable for “general” or “universal” biotyping. They are all found in the same small cluster far beyond the other two groups of *Tribolium CSP* genes. Some of the genes have the intron exactly of the same length. Their intron is always located at the same position, i.e., after residue 45 (K or E), and if there is a variation, it varies very little from 48 to 211 bps (Figure 2). Such very short introns may require involvement of highly specific mechanisms for correct slicing. The minimum functional intron length in insects and plants is between about 38–73 bps [54,55]. Therefore, the minimum functional intron length in CSP genes should be investigated in detail, in particular in beetles. In addition of short conserved elements, this could reveal a novel pre-mRNA splicing pathway [56]. A lysine-to-glutamic acid residue switch occurred in the exon/intron boundary at position 45 in *AAJJ0012J*, but this did not change border splice site (Figure 2). So, not only genes such as *AAJJ1796A* and *BmorCSP19*, but also the border splice sites of *CSPs* could be used for “universal signal” of genosensing in a new development of typing methods.

## 3. Biosensing Expression of Tissue-Specific RNA Mutations

**Figure 3:**
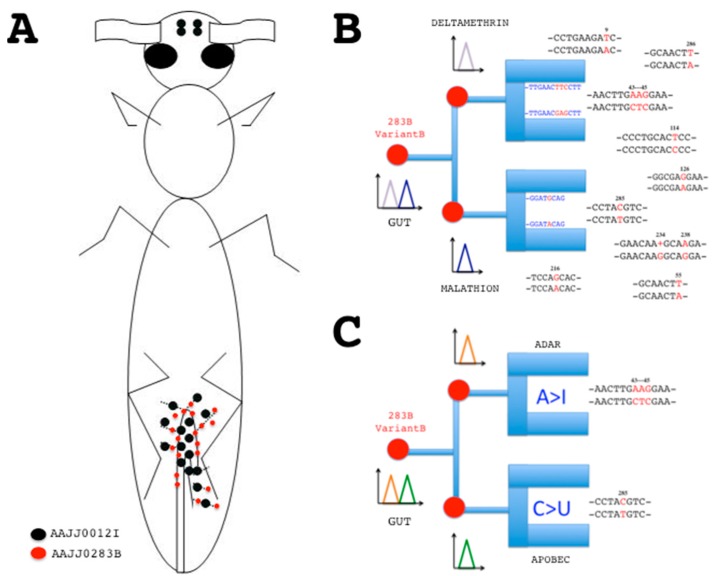
Principle of sensor chips based on CSP-RNA mutations. (**A**) Distribution of EST-cDNA sequences (red/black dots) encoding CSPs in young adults of *T. castaneum* (flybase.org). Hindgut and Malpighian tubules: DT776391, DT782506, DT783721, DT785607, ES548720, ES548948, ES549909, ES549995, ES550112, ES550327, ES550345, ES550411, ES550426, ES550567 (*AAJJ0012I*); DT773281, ES548660, ES548870, ES549463, ES549545 (*AAJJ0283B*). Head: ES544614, ES544679 (*AAJJ0012I*) [56,57]; (**B**) Electrochemical DNA/RNA hybridization sensors based on CSP mutant sequences. Specific base mutation strands will attach luminescent nucleic acid probes immobilized on the chip, revealing tissue-specific RNA mutations at a given time and function. The probes correspond to CSP sequences with and without mutations. Gut RNA samples are applied on the chip. Signal for CTC mutations at position 43–45 of AAJJ0283B is indicative of deltamethrin infection, while signal for C-to-U mutation at position 285 is indicative of malathion exposure; (**C**) RNA editing enzyme-linked electrochemical fluorescent mutation biosensor chip. Instead of DNA probe, ADAR binding site will trap A > I base mutations, while apobec binding site will depict C > U base mutations. Base pairing interaction or activation of enzyme binding site recruit the RNA single point mutation to the biosensor surface, prelude to signal transduction. The sum of the two fluorescent signals is diagnosis for the presence and combinatorial action of specific point mutations and/or small variants on an RNA strand coding for a given protein isoform at a particular development stage in a cell organ tissue site. Mutation sequences (RNA variants) are for CSP gene *AAJJ0283B* (see Table S1). Single point mutations are shown in red.

Gene structure including border splice site is not the only common element comparing *CSPs* between *Bombyx* and *Tribolium*. Another important common feature is found in the expression of CSP genes, in particular in its regulation via RNA editing. This could be extremely useful information to make a genetic or enzyme chip for detection of cell and tissue-specific mutations (Figure 3).

RNA variance and protein diversity have been originally described in *B. mori* CSPs [57,58,59,60,61,62,63]. Similarly, about twelve tag sequences (DT787080-EC010944; *e*-value: 11.76938e-180 – 1.36145e-159) can be found in the EST database of the beetle *T. castaneum* for gene encoding AAJJ0283B and variants [64,65], suggesting that RNA editing is a common feature of CSP gene expression in insects. TcasCSP-RNA variants correspond to many different single base substitution mutations including not only A-to-G or U-to-C replacements, but also a large number of less conventional substitutions such as U-to-A or G, C-to-U, G-to-A or G insertion as described for BmorCSPs [57,58,59,60,61,62,63,64,65]. Interestingly, in *Tribolium*, some of these mutations (U-to-A and C-to-U) linked to CSP *nt* sequences remain silent, but most of them lead to a change in one amino acid in the protein, particularly in the N-terminus (D-to-E switch at position 3, K-to-L switch at position 15) [64,65]. The nucleotide sequence from JF08 *T. castaneum* cDNA clone 511225 (467 bps) displays stop codon mutation and a completely changed motif at the C-terminal end, possibly resulting in a CSP protein with a novel folding [58,63,64,65]. Therefore, the potential applications of *CSPs* are genetic chips not only for genomic analysis and biotyping, but also for RNA analysis and typing of a set of mutations crucial for cell tissue function (Figure 3) [57,58,59,60,61,62,63,64,65].

RNA editing ought to regulate gene expression in a versatile protein family such as CSPs, which could be extremely useful information in a search for genosensors and/or biosensors based on multi-gene family, multi-function and development/tissue-dependent RNA variance in insects. Distribution of EST-cDNAs encoding CSPs in the red flour beetle *T. castaneum* is in strong agreement with insecticide xenobiotic up-regulation in *quasi* all *CSPs* from most tissues of the adult insect body, particularly the gut (Figure 3A) [29,66]. The analysis of the currently available *Tribolium* EST database shows that *AAJJ0330A* (CB334791–CB337098) and *AAJJ0012G* (CB33646–CB336463) are highly expressed in the whole body of *tribolium* at the embryonic stage, refuting an olfactory function for these genes [67,68].

In young adults, ESTs for *T. castaneum “CSPs”* such as *AAJJ0012I* and *AAJJ0283B* express in the hindgut and Malpighian tubules, the chief organs of excretion in the digestive system of the insect, refuting an olfactory function also for these two *Tribolium* CSP genes (Figure 3A). Hindgut and renal tubules in insects are essential for digestion and innate immunity, in particular in the degradation of pathogens that may have escaped the midgut immune system [69,70]. The TcasCSP gene *AAJJ0269C* is strongly expressed in the carcass of immune-challenged late larva after removal of the alimentary channel (ES547313, ES547314, ES547742, ES547743, ES548097), emphasizing the functional importance of CSPs not in olfaction, but in the immune response as described in flies, moths and whiteflies [14,16,27,29]. In the insect immune system from the early embryonic to the senescent adult stages, the high diversity of CSP RNA editing isoforms may be important to breakdown, metabolize and transport a broad diversity of residues, wastes, antigenic variants and/or variety of toxic xenobiotic chemical substances, perhaps in strong association with various strains of beneficial bacteria from the insect gut flora.

Pinpointing variation sites over the RNA coding sequence of *CSPs* could be therefore very helpful to mark slowly the activity of a tissue, in particular in the gut (Figure 3). Using genetic chips for insect biotype recognition is described in tephritid flies and mosquitoes [71,72]. In human, DNA chips are widely used for the detection of polymorphisms or mutations in genetic pathologies such as hemophilia [73,74]. Using *CSPs*, we are not dealing just with another type of DNA chip for genetic mutations. We are dealing with a new type of RNA sensors to be developed not for biotype, disease or malfunction, but for detection of mutation sequences associated with function changes in a particular tissue or cell type. RNA mutation detection is technically feasible by using conducting nanoparticles or polymers combined to a process of enzyme-amplified electronic transduction or any colorimetric electrochemical measurement of nanoscale interactions between the mutant RNA targets in solution, a DNA recognition probe and an electrode signal (Figure 3B) [75,76,77,78]. It could use, for instance, the quartz crystal microbalance detection based on monobase-coded cadmium tellurium nanoprobe, a fluorogenic ribonuclease protection assay, a principle of localized surface plasmon resonance (LSPR), Biacore or some other applied principle of nucleotide sensor chips [79,80,81,82,83]. These nanoprobes and/or chips applied to human health aim to help medical research to understand how the mutations in specific proteins predispose to syndrome [84]. Here, we aim to help agricultural research by developing specific nanoprobes and/or chips applied to insect cell biology in order to understand how RNA mutations in a multifunction protein-coding gene family such as *CSPs* allow new molecular recognition sites in a particular tissue (e.g., the gut) under various normal (and abnormal) physiological conditions. Detection of a particular mutation signal (A-to-I or C-to-U) in a gut CSP such as AAJJ0283B may be indicative of the degradation of a specific xenobiotic chemical such as deltamethrin or malathion (Figure 3B). A CSP mutation could thus be used as a very efficient and reliable biological indicator of environmental pollution for a very specific insecticide substrate.

Sensing CSP mutations may be also important to explore basic aspects of pheromone research. Xuan et al. have reported that the RNA editing process of *BmorCSPs* is tissue-dependent with a lipid spring, the female moth pheromone gland, as critical primary source of edited variants [57]. This strongly suggests that there is a strong DNA or RNA-dependent RNA polymerase activity (DdRp or RdRp) extended not only across tissues involved in lipid metabolism and/or xenobiotic degradation, but also across tissues specifically involved in sex pheromone biosynthetic pathways [57,58,59,60,61,62,63]. These properties of editing enzymes such as functional inference and tissue-specificity may help build the active sites of a recognition layer in a chip tuned to specific RNA base mutations, instead of a nucleotide probe (Figure 3C). RNA editing enzymes largely refer to adenosine deaminases acting on RNA (ADARs) and apolipoprotein B mRNA editing enzyme (apobec) catalytic polypeptide-like that mediate A-to-I and C-to-U base substitution, respectively [85,86,87,88,89]. How ADAR and/or apobec bind to RNA and what is the functional structure of enzyme-RNA complexes are questions to answer for evolutionary, mechanistic, fundamental and applied aspects of RNA mutations [90,91,92,93,94,95]. One of the possible applied aspects of ADAR and apobec characterization could refer to mutation sensors, i.e., design a functional matrix immobilized on a chip in order to trap specific RNA motifs (Figure 3C). This could be particularly relevant to detect in a given organism with specific traits a typo-single nucleotide base mutation crucial for adaptation, development, function and/or evolution in some particular cells. Construction and characterization of ADAR and/or apobec as recognition elements of RNA chips for detection of base mutations could illuminate for instance which specific target organ or group of tissues bear the mutation and when, comparing eventually mutation analysis under physiological and toxicological conditions. It could also compare genomic and RNA templates, investigating evolutionary and functional aspects of exon (coding) versus intron (non-coding) RNA editing or how fast a non-silent base mutation can occur and drastically affect the proteome repertoire [96,97]. A cytidine deaminase edits C to U to permit proper folding and functionality of RNA in archaean bacteria such as *Methanopyrus kandleri* [98]. A-to-I transition in the eukaryote brain is considered as crucial for cognitive processes influencing learning and memory [99]. The study of naturally occurring mutation modifications of messenger RNAs as found for *CSPs* is much more than about a new genetic biosensor. It could help build a complete tool for A-to-I, C-to-U and/or many other types of mutation analysis, underlying species, strain or genotype differences in expression of one particular gene in a specific type of cell, a main tissue type, a major complex organ or a complete functional organismal system [57,58,59,60,61,62,63,100].

## 4. Protein Structures for Multi-Lipid and Multi-Insecticide Sensors

The multitude of tasks achieved by CSPs is particularly useful to design a great variety of sensor chips to be exploited by many industries as important and diverse as medicine, agriculture and environment protection.

**Figure 4:**
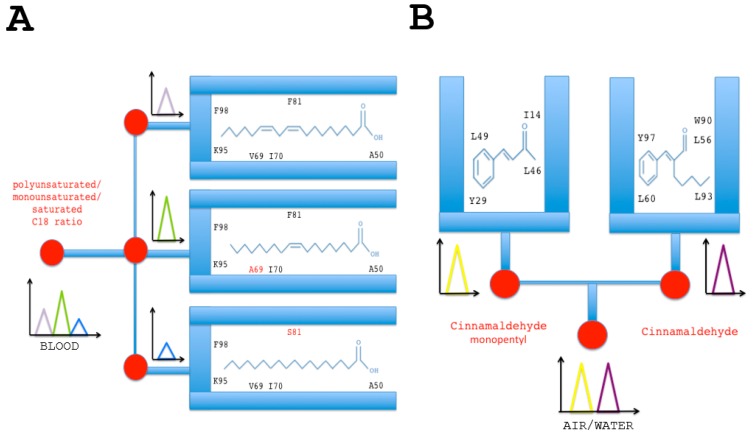
Lipid and insecticide (cinnamaldehyde) chemo-sensor chips based on functional group binding sites of *B. tabaci* CSPs. (**A**) Sensors based on the interaction of CSP protein with lipids. Specific mixtures of polyunsaturated (linoleic acid), monounsaturated (oleic acid) and unsaturated (stearic acid) lipids will attach luminescent variant CSP protein isoform probes immobilized on the chip. Amino acid in red indicates specific missense mutation in the binding site of BtabCSP1 [14,16]. Lipid-CSP pairing interaction and activation of variant functional binding sites recruit the various forms of fatty acid (polyunsaturated, monounsaturated and saturated) to the biosensor surface, prelude to signal transduction. The sum of the three fluorescent signals is diagnosis for the presence and concentration of each lipid species, thereby providing the lipid basis along with the ratio of the total lipid composition in any relevant biological sample; (**B**) Sensors based on the interaction of CSP with cinnamaldehyde and a cinnamaldehyde chemical derivative (cinnamaldehyde monopentyl). The functional binding sites immobilized on the insecticide chip are those identified for BtabCSP2 and BtabCSP3 [16]. The signals diagnose the presence of different combinations of cinnamaldehydes and measure their levels in the environment.

The role of CSP in lipid transport has been demonstrated by Liu et al. for a protein ubiquitously expressed in the *Bemisia* body, highly expressed in field strains of Q-biotype, and up-regulated over insecticide thiametoxam exposure, i.e., BtabCSP1 [14,16]. BtabCSP1 protein does not directly interact with the neonicotinoid insecticide molecule, but rather with a major constituent of cellular lipids, namely linoleic acid (C18:2 n-6) [16]. Interestingly, a number of nucleotide variations have been described at the RNA level underlying CSP1 genotype. A-to-I, U-to-C and C-to-U as well as insertion mutations have been found for *B. tabaci CSP1*, and these missense mutations can possibly result in sex and/or tissue-associated protein variants [14,16]. Lipid-protein interaction and RNA variance in BtabCSP1 may convey the idea of using the protein and the protein variants to compose a matrix particularly suitable for the simultaneous detection of multiple lipid species (Figure 4A). The principle of multi-C18 lipid detectors is based on the functional binding sites identified for *B. tabaci* CSP1 protein (Figure 4A) [16]. Alanine at position 50 (A50) anchors the acid group, while the tip of the long hydrophobic chain curves to attach with Lysine 95 and Phenylalanine 98, leaving the double bonds of linoleic acid sandwiched between Val69, Ile70 and Phe81 [16] (Figure 4A). A specific missense pinpoint single base mutation leads to change Valine residue to Alanine at position 69 or Phenylalanine to Serine at position 81, resulting in both cases in a functional switch of the CSP1 protein binding site (Figure 4A) [14,16]. A69 mutant switches to bind monounsaturated oleic acid, while S81 protein variant switches to bind saturated octadecanoic (stearic) acid, helping us move forward with the idea of developing electroluminescent or thermoelectric C18-lipid sensors based on multi-element variants of CSP1 protein structure (Figure 4A).

Other different categories of liposensors or lipometers could exploit and value to a great vast extent the remarkable capacity of CSPs to solubilize fatty acid lipids [16,101,102,103,104]. Combining CSPs with other fatty acid-binding proteins such as THP12 or Niemann-Pick type C2 could even give an infinite dimension to such a new family of lipid protein sensor chips [105,106]. This would certainly be a highly valuable method for determining the composition and concentration of lipids as well as the lipid ratio in microorganisms, transgenic plants, glands, oils or blood samples. This would also be of a particularly high interest approach for chemical ecology and the analysis of waxes and cuticular blends that constitute part of inter- and intraspecific chemical signatures in many species of gregarious social insects [107,108,109]. Similarly, analysis of fatty acid and lipid composition and their changes in arthropod/crustacean species or vertebrate animals with relation to different organs and tissues would be essential for understanding on environment adaptation, climate change or fundamentally conserved evolutionary traits [110]. A lipometer based on functional binding sites of CSP-Thp12-Niemann Pick type C2 proteins would be particularly useful to measure lipid dynamics in neurons during renewal over lesion, wound, growth, pheromone sensing, flight activity, migratory paths and/or any lipidemia change following a physiological response to food uptake, diet, temperature stress, chemical contamination or CO_2_ exposure. For instance, the information about mobilization and storage of lipid components would be valuable from an evolutionary point of view in scorpions that are living species with very primitive traits, which have varied minimally through time [111]. The information about lipid accumulation in insect and/or bacterial species that have developed whole pesticide resistance and/or disease transmission capacity would provide the same relevant knowledge for control of major agricultural or horticultural pest species as well as strains of mosquitoes or ticks that are vectors of human pathogens [112,113]. Finally, a CSP-based lipid sensor would be particularly relevant in medicine not only for the diagnosis of hyperlipidemia associated to diabetes and/or various functional heart diseases, but also for the checkup examination of lipid levels over therapeutic treatment [114].

Alternatively, CSPs and/or other binding proteins could be very useful to build sensor chips tuned to specific categories of toxic pesticide molecules, opening a new issue in the evaluation of air and environment chemical pollution. This comes out after the finding from Liu et al.’s study in which additional *Bemisia* CSPs, BtabCSP2 and BtabCSP3, show a preferential affinity not for lipids, but rather for a certain class of small toxic volatile chemicals such as cinnamaldehydes [16]. This study demonstrates that, while some CSPs play a pivotal role in transport of lipid molecules in general, innate, in-born and/or non-specific immunity response to xenobiotics infection, some other CSP proteins are more directly involved in the capture of the xenobiotic compounds, prelude to their enzymatic degradation by the acquired, adaptive, humoral cell-mediated and/or much more specific immunological system [16]. The binding affinities determined in *B. tabaci* CSP proteins could be therefore of a particular interest to help develop two different types of ligand biosensors: (1) lipid sensors and (2) insecticide sensors (Figure 4). The immobilization of cinnamaldehyde or its biological derivatives-binding sites as those identified for BtabCSP2 and BtabCSP3 could well serve as a reference to design a protein chip of the future, analyzing not only bark tree-plant oil composition, cream, perfume or liquor aromatization, but also food, soil and water contamination (Figure 4B) [16,115,116]. Cinnamaldehyde is known to be carcinogenic, mutagenic and/or teratogenic for many various organisms from bacteria to human, urging to find new ways of analytical study on this chemical. This is particularly mandatory since cinnamaldehyde is used as a rubber reinforcing agent, a cleaner substance for iron metals, an ingredient of cigarettes, a pet repellent and an attractant for common home insect pests (termites), among many others. Such widespread use and constant daily exposure lead to the importance of tracking and balancing environment and pool water for cinnamaldehyde. The multifunctional properties described for *Bemisia* CSPs demonstrate the pivotal importance of this protein family for biosensing [16]. BtabCSP1 protein chips will help build efficient lipid sensors, while BtabCSP2 and BtabCSP3 protein chips will be extremely useful to test levels of toxic compounds such as cinnamaldehyde and cinnamyl derivatives (Figure 4) [16].

Whether many various CSPs can be used for insecticide detection needs further investigations after Liu et al. and Xuan et al. [14,16,29]. CSPs are important molecules that can directly act to reduce the effect of xenobiotics, but it is so far restricted to some xenobiotics only (avermectins, neonicotinoids and plant oil chemicals) [14,16,29]. Other different types of BtabCSPs and/or binding proteins may bring some further components in the bio-sensing analysis of xenobiotics or chemical groups of xenobiotics. This is true at least for a candidate in the odor binding protein (OBP) family [117]. Therefore, it is highly probable that CSPs (and OBPs) are general secondary binders that are involved in many resistance cases. It is unlikely that they are just part of a consequence mechanism rather than a direct primary mechanism against xenobiotic infection. Up-regulation of *CSPs* upon bacterial/viral infection and/or insecticide exposure (abamectin and thiametoxam), binding to xenobiotic compounds as well as the expression of both *CSPs* and *OBPs* in prokaryotes (no nerve cells) strongly support a fundamental role of this gene family in the insect (and bacterium) immunological responses [14,16,27,29,30]. In the whitefly *B. tabaci*, *CSP1*, *CSP2* and *CSP3* represent homologous genes with about 40% identity with each other. All the three genes appear to be essential in the immune response, although not at the same level [16]. So, the lack of cross-resistance between different families of insecticides in field strains of the whitefly *B. tabaci* may strongly suggest that exposure to other classes of environmental xenobiotic compounds involve other different types of CSPs and/or binding proteins, expanding thereby in a drastic manner the catalogue or the list of protein biosensors tuned to insecticide chemicals [14,16,29,118]. It does not mean that CSPs (and OBPs) are the only involved in resistance and cross-resistance mechanisms and resulting phenotypes. Other families such as the well-known cytochrome P450 monooxygenases, glutathione S-transferases or esterases are important detoxification enzymes that can act in combination with the CSPs [29]. In some cases, against some molecules such as bacterial monooxygenases and organochlorides, they might even be the major actors while the binding proteins (CSPs and OBPs) are secondary/complementary molecular components.

Producing CSP-based biosensors to detect environmental pollution is not just an idea. Biosensor arrays based on immobilization of OBPs onto transducers such as a quartz crystal microbalance platform or a reduced grapheme oxide field-effect transistor make it feasible [119,120]. Focusing on a multifunction binding protein family such as CSPs specifically has potential to bring the biosensor technology and assay design to understand basic principles of cell and tissue biology as well as towards more applied aspects in the analysis of the environment change and its impact on human health. The conclusion of our review on *CSPs*, the utility and mechanism of sequence variation in this gene family as well as the binding properties of the proteins they encode, is their usefulness not only for identifying insect pest biotypes, but also in biosensors for lipids and toxic chemicals. The 3-part overview (gene, RNA and protein) provides the insight into applications of CSP gene family in both applied and basic science and a particular case of multi-point perspective in which a novel thinking on strategies to mutation sensing in insects may be the most interesting.

## Supplementary Materials

The following are available online at www.mdpi.com/1424-8220/17/8/1801/s1, Table S1: Repertoire of 19 CSP sequences identified by in silico analysis of the beetle genome database (BeetleBase, http://www.hgsc.bcm.tmc.edu). * Length (in bps) corresponds to the coding region of the gene from start (ATG) to stop codon (TAA).
