# Supplementary material for: Genotyping and Bio-Sensing Chemosensory Proteins in Insects
Source: Sensors (Basel). 2017 Aug 4;17(8):1801. doi: 10.3390/s17081801 (PMC5579523; doi:10.3390/s17081801)
Supplement: Supplementary file 1 [file sensors-17-01801-s001.pdf]

sensors

# Genotyping and Bio-Sensing Chemosensory Proteins in Insects

**Guoxia Liu, Philippe Arnaud, Bernard Offmann and Jean-François Picimbon**

**Table S1.** Repertoire of 19 CSP sequences identified by *in silico* analysis of the beetle genome database (BeetleBase, http://www.hgsc.bcm.tmc.edu). * Length (in bps) corresponds to the coding region of the gene from start (ATG) to stop codon (TAA).

| Gene name | Length * | n introns | Scaffold Nb | Acc. Nb (EST) |
|---|---|---|---|---|
| *AAJJ0012A* | 406 | 1 | AAJJ01000012.1 | ABH88191 |
| CSP03 | | | | ES549186 |
| TcasCSP18 | | | | NM_001045821 |
| TcCSP7R | | | | NP_00139286.1 |
| | | | | XM_967752 |
| *AAJJ0012B* | 406 | 1 | AAJJ01000012.1 | ABH88190 |
| CSP04 | | | | NM_001045819 |
| TcasCSP17 | | | | NP_001039284.1 |
| TcCSP7Q | | | | XM_967624 |
| *AAJJ0012C* | 413 | 1 | AAJJ01000012.1 | ABH88182, ABH88189 |
| CSP05 | | | | NM_001045818 |
| TcasCSP9 | | | | NP_001039283.1 |
| TcasCSP16 | | | | XM_967584 |
| TcCSP7P | | | | XM_008197998.2 |
| *AAJJ0012D* | 478 | 1 | AAJJ01000012.1 | ABH88188 |
| CSP08 | | | | NM_001045826 |
| TcasCSP15 | | | | NP_001039291.1 |
| TcCSP7O | | | | |
| *AAJJ0012E* | 413 | 1 | AAJJ01000012.1 | ABH88187 |
| CSP02 | | | | NM_001045817 |
| TcasCSP14 | | | | NP_001039282.1 |
| TcCSP7N | | | | XM_967541 |
| *AAJJ0012F* | 406 | 1 | AAJJ01000012.1 | ABH88186 |
| CSP06 | | | | NM_001045816 |
| TcasCSP13 | | | | NP_00139281.1 |
| TcCSP7M | | | | XM_967497 |
| *AAJJ0012G* | 416 | 1 | AAJJ01000012.1 | ABH88185 |
| CSP09 | | | | AJ973445.1 |
| ORF2 | | | | CB336464 |
| TcasCSP12 | | | | NM_001045815 |
| TcCSP7L | | | | NP_001039280.1 |
| | | | | XM_967442 |
| *AAJJ0012H* | 416 | 1 | AAJJ01000012.1 | ABH88184 |
| CSP01 | | | | NM_001045814 |

| | |
|---|---|
| TcasCSP11 | NP_001039279.1 |
| TcCSP7K | XM_967394 |

**Table S1**. *Cont*.

| | | | | |
|---|---|---|---|---|
| *AAJJ0012I*<br>CSP07<br>TcasCSP10<br>TcCSP7J | 422 | 1 | AAJJ01000012.1 | ABH88183<br>DN650811-ES550426<br>NM_001045813<br>NP_001039278.1<br>XM_967340 |
| *AAJJ0012J*<br>LOC103313838 | 327 | 1 | AAJJ01000012.1 | XM_008198119 |
| *AAJJ0269A*<br>CSP11<br>TcasCSP4<br>TcCSP7G | 436 | 1 | AAJJ01000269.1 | ABH88177<br>NM_001045820<br>NP_001039285<br>XM_967744 |
| *AAJJ0269B*<br>CSP14<br>TcasCSP5<br>TcCSP7F | 471 | 1 | AAJJ01000269.1 | ABH88178<br>NM_001045822<br>NP_001039287<br>XM_967788<br>XM_015981290 |
| *AAJJ0269C*<br>CSP13<br>TcasCSP6<br>TcCSP7E | 819 | 1 | AAJJ01000269.1 | ABH88179<br>DT794283-ES548097<br>NM_001045823<br>NP_001039283<br>XM_967831<br>XM_008195554 |
| *AAJJ0269D*<br>CSP12<br>TcasCSP7<br>TcCSP7D | 416 | 1 | AAJJ01000269.1 | ABH88180<br>NP_001039289<br>NM_001045824<br>XM_967868<br>XM_008195555 |
| *AAJJ0269E*<br>CSP16<br>TcasCSP8<br>TcCSP7C | 325 | 1 | AAJJ01000269.1 | ABH88181<br>NM_001045825<br>NP_001039290<br>XM_967938 |
| *AAJJ0283A*<br>TcasCSP19<br>TcCSP7A | 390 | 1 | AAJJ01000283.1 | ABH88192<br>NM_001045811<br>NP_001039276<br>XM_965061 |
| *AAJJ0283B*<br>CSP18<br>TcasCSP20<br>TcCSP7B | 406 | 1 | AAJJ01000283.1 | ABH88193<br>DN651210-ES551268<br>G6824.81<br>NM_001045809<br>NP_001039274<br>XM_961519<br>XM_008201895 |

**Table S1**. *Cont*.

| | | | | |
|---|---|---|---|---|
| *AAJJ0330A* | 399 | 0 | AAJJ01000330.1 | ABH88176 |
| CSP20 | | | | CAJ01491 |
| TcasCSP2 | | | | CB334791- CB337098 |
| TcCSP3A | | | | DQ855489 |
| | | | | NM_001045812 |
| | | | | NP_001039277 |
| | | | | XM_967323 |
| *AAJJ1796A* | 367 | 2 | AAJJ010001796.1 | ABH88175 |
| CSP17 | | | | DN651433-EC010405 |
| TcasCSP1 | | | | NM_001045808 |
| TcCSP2A | | | | NP_001039273 |
| | | | | XM_008202712 |
